# Inguinal Herniation of the Urinary Bladder Presenting as Recurrent Urinary Retention

**DOI:** 10.1155/2015/531021

**Published:** 2015-05-17

**Authors:** Amit Frenkel, Aviel Roy-Shapira, Ilan Shelef, Gadi Shaked, Evgeni Brotfain, Leonid Koyfman, Abraham Borer, Moti Klein

**Affiliations:** ^1^General Intensive Care Unit, Soroka University Medical Center and the Faculty of Health Sciences, Ben-Gurion University of the Negev, 84101 Beer-Sheva, Israel; ^2^Department of General Surgery, Trauma Unit, Soroka University Medical Center and the Faculty of Health Sciences, Ben-Gurion University of the Negev, 84101 Beer-Sheva, Israel; ^3^Department of Radiology, Soroka University Medical Center and the Faculty of Health Sciences, Ben-Gurion University of the Negev, 84101 Beer-Sheva, Israel; ^4^Infection Control and Hospital Epidemiology Unit, Soroka University Medical Center and the Faculty of Health Sciences, Ben-Gurion University of the Negev, 84101 Beer-Sheva, Israel

## Abstract

Herniation of the urinary bladder into the inguinal canal is an uncommon finding, observed in 0.5–4% of inguinal hernias (Curry (2000)). It is usually associated with other conditions that increase intra-abdominal pressure such as bladder neck obstruction due to prostatic hypertrophy. Consequently, in men, it is usually associated with some degree of urinary retention. We present a 42-year-old man in whom herniation of the urinary bladder was the cause of urinary retention, and not vice versa. The patient was on tumor necrosis factor alpha antagonist (TNFA) (Etanercept) for severe Ankylosing spondylitis. Initially, the urinary retention was thought to be a side effect of the medication, but after the drug was discontinued, urinary retention persisted. CT and MRI demonstrated huge herniation of the urinary bladder into the inguinal canal. Immediately after the hernia was repaired, bladder function was restored. TNF treatment was restarted, and no further urinary symptoms were observed in the next two years of follow-up. In this case, the primary illness and its treatment were distracting barriers to early diagnosis and treatment. In younger patients with a large hernia who develop unexpected urinary retention, herniation of the urinary bladder should be highly considered in the differential diagnosis.

## 1. Introduction

Herein we present an unusual case of urinary tract obstruction caused by herniation of the urinary bladder into the inguinal canal. While some herniation of the bladder into the inguinal canal can be seen in up to 4% of hernias [1], most reported cases occur in elderly men and are associated with greater or lesser degree of distal urinary tract obstruction, most often due to an enlarged prostate; increased abdominal pressure due to bladder neck obstruction is a common reason for elderly patients to seek surgical consultation for a previously asymptomatic inguinal hernia.

In the case presented, the herniation of the urinary bladder into the inguinal canal was the cause of urinary retention rather than vice versa. The diagnosis was made in a rather obtuse way, and the purpose of the presentation is to alert physicians to this possibility.

## 2. Case Presentation

A 42-year-old man with a long history of Ankylosing spondylitis presented with acute urinary retention three months after he was started on a TNF alpha antagonist.

The patient was well until, at the age of 25, he begun to complain of inflammatory back pain due to Ankylosing spondylitis. Initially, he responded well to NSAID medication, at least symptomatically, but the disease progressed over the years, leading to increasing flexion deformity of the neck, thoracic kyphosis, and loss of normal lumbar lordosis. Due to decreasing clinical response coupled with marked elevation of CRP, he was placed on Etanercept (Enbrel), a tumor necrosis factor alpha antagonist (TNFA) [2].

Treatment with Etanercept had a dramatically beneficial effect on the quality of life of the patient. Clinically, the back pain waned to nothing, and overall mobility was improved while CRP normalized. However, three months after the treatment was initiated, the patient started having obstructive urinary symptoms: stress incontinence, dysuria, staining to void, nocturia, and increased frequency of passing small quantities of urine. The symptoms culminated in complete distal urinary tract obstruction and required catheterization with evacuation of 1200 mL of residual urine. There was no evidence of urinary tract infection, both by culture and by direct examination; an ultrasonic examination of the urinary tract was normal. There was no evidence of bladder stones.

Alpha blockers were started empirically, and the catheter was removed 72 hours later, with some relief, but the patient continued to have difficulties with micturition and had “two-stage urination” (a normal first stream, followed by a protracted and hesitant stream).

Since there was no evidence of physical bladder neck obstruction, the differential diagnosis at this stage included a side effect of TNFA and possibly cauda equina syndrome, which is a rare complication of Ankylosing spondylitis [3].

**Figure 1:**
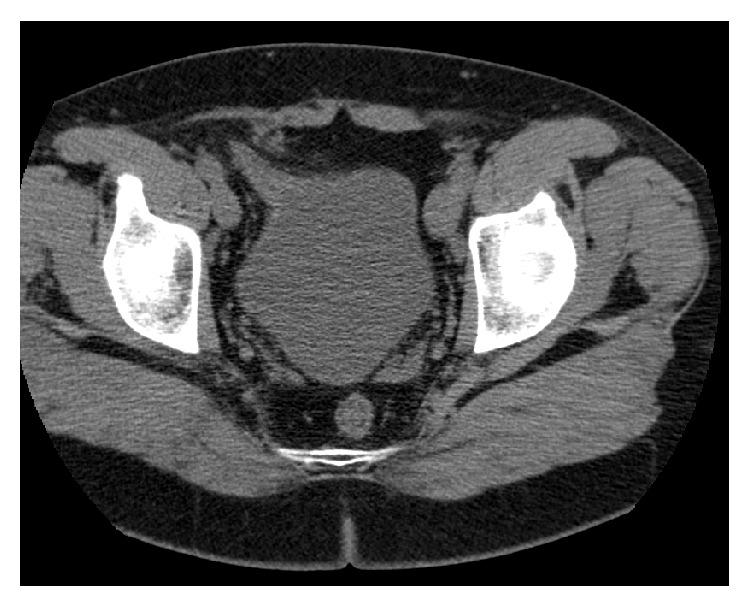
Noncontrast CT: right inguinal hernia containing the bladder wall.

Consequently, Etanercept was discontinued, and the patient was referred to CT scan and MRI of the spinal column. After TNFA was stopped there was no improvement in the urinary symptoms; in fact, complete UTO recurred, and the patient had to be catheterized for a second time. Cauda equina syndrome was ruled out, but the noncontrast CT (Figure 1) revealed right inguinal hernia containing the bladder wall and distorted the anatomy of the bladder neck.

The patient was referred to surgery. The urinary bladder herniation was extraperitoneal, alongside an indirect hernia sac, and easily reducible. Tension-free repair of the hernia was performed successfully.

The postoperative course was uneventful. Soon after surgery, the urinary catheter was removed with no recurrence of urinary retention, and TNFA was restarted. Over two years of follow-up, the patient continued to enjoy the benefits of TNFA treatment with no further urinary symptoms.

## 3. Discussion 

The great majorities of significant herniations of the gallbladder occur in elderly men and are likely to be the result of the need to increase contraction force to overcome distal urinary tract obstruction due to an enlarged prostate [4]. Most of the time, herniation of the bladder does not cause any symptoms, since only a minimal portion of the bladder is involved.

As in this case, the bladder usually herniates extraperitoneally, along the hernia sac (paraperitoneal). Rarely, the inguinal part of the bladder appears to be fully encased by peritoneum (intraperitoneal). It can herniate without a concomitant peritoneal sac and present as a femoral hernia or obturator hernia.

Urinary retention episodes associated with herniation of the bladder have been reported and are often indistinguishable from symptoms of other causes of distal UTO: hematuria, hesitancy, frequency, burning sensation, frequent infections, or cystolithiasis.

This case is unusual because the urinary symptoms occurred in a relatively young man and were not associated with physical bladder neck obstruction. In the majority of cases, patients seek surgical consultation because increased abdominal pressure during micturition enlarges a previously asymptomatic inguinal hernia or causes abdominal contents to protrude through the fascial defect, and the involvement of the urinary bladder is discovered serendipitously during the hernia repair operation.

In retrospect, the hernia was palpable but erroneously considered asymptomatic. Since “two-stage urination” is a common feature of significant herniation of the urinary bladder, it should have provided a clue to the correct diagnosis; however, the associated illness was a distracting factor and delayed the diagnosis and the operation, at the cost of interrupting the treatment of the primary condition and considerable patient discomfort.

We conclude that although rare herniation of the urinary bladder should be suspected in any patient who presents with unexpected signs of UTO, particularly when “two-step urination” is observed.
