# Corrigendum to “Inguinal Herniation of the Urinary Bladder Presenting as Recurrent Urinary Retention”

**DOI:** 10.1155/2019/8219316

**Published:** 2019-10-14

**Authors:** Amit Frenkel, Aviel Roy-Shapira, Ilan Shelef, Gadi Shaked, Evgeni Brotfain, Leonid Koyfman, Abraham Borer, Moti Klein

**Affiliations:** ^1^General Intensive Care Unit, Soroka University Medical Center and the Faculty of Health Sciences, Ben-Gurion University of the Negev, 84101 Beer-Sheva, Israel; ^2^Department of General Surgery, Trauma Unit, Soroka University Medical Center and the Faculty of Health Sciences, Ben-Gurion University of the Negev, 84101 Beer-Sheva, Israel; ^3^Department of Radiology, Soroka University Medical Center and the Faculty of Health Sciences, Ben-Gurion University of the Negev, 84101 Beer-Sheva, Israel; ^4^Infection Control and Hospital Epidemiology Unit, Soroka University Medical Center and the Faculty of Health Sciences, Ben-Gurion University of the Negev, 84101 Beer-Sheva, Israel

In the article titled “Inguinal Herniation of the Urinary Bladder Presenting as Recurrent Urinary Retention” [1], there was an error in the Discussion, which should be corrected as follows:

“The great majorities of significant herniations of the bladder occur in elderly men and are likely to be the result of the need to increase contraction force to overcome distal urinary tract obstruction due to an enlarged prostate [4].”
